# Distinct functional consequences of ECEL1/DINE missense mutations in the pathogenesis of congenital contracture disorders

**DOI:** 10.1186/s40478-017-0486-9

**Published:** 2017-11-13

**Authors:** Kenichi Nagata, Mika Takahashi, Sumiko Kiryu-Seo, Hiroshi Kiyama, Takaomi C. Saido

**Affiliations:** 1 Laboratory for Proteolytic Neuroscience, RIKEN Brain Science Institute, Saitama, 351-0198 Japan; 2 Department of Functional Anatomy and Neuroscience, Nagoya University, Graduate School of Medicine, 65 Tsurumaicho, Showa-ku, Nagoya, 466-8550 Japan

**Keywords:** Distal arthrogryposis, ECEL1, DINE, Motor nerve, Axon guidance, Neuromuscular junction

## Abstract

Endothelin-converting enzyme-like 1 (*ECEL1*, also termed *DINE* in rodents), a membrane-bound metalloprotease, has been identified as a gene responsible for distal arthrogryposis (DA). *ECEL1*-mutated DA is generally characterized by ocular phenotypes in addition to the congenital limb contractures that are common to all DA subtypes. Until now, the consequences of the identified pathogenic mutations have remained incompletely understood because of a lack of detailed phenotypic analyses in relevant mouse models. In this study, we generated a new knock-in mouse strain that carries an *ECEL1/DINE* pathogenic G607S missense mutation, based on a previous study reporting atypical DA hindlimb phenotypes in two siblings with the mutation. We compared the morphological phenotypes of G607S knock-in mice with C760R knock-in mice that we previously established. Both C760R and G607S knock-in mouse embryos showed similar axonal arborization defects with normal trajectory patterns from the spinal cord to the target hindlimb muscles, as well as axon guidance defects of the abducens nerves. Intriguingly, distinct phenotypes in DINE protein localization and mRNA expression were identified in these knock-in mouse lines. For G607S, DINE mRNA and protein expression was decreased or almost absent in motor neurons. In the C760R mutant mice DINE was expressed and localized in the somata of motor neurons but not in axons. Our mutant mouse data suggest that *ECEL1/DINE* G607S and C760R mutations both lead to motor innervation defects as primary causes in *ECEL1*-mutated congenital contracture disorders. However, the functional consequences of the two mutations are distinct, with loss of axonal transport of ECEL1/DINE in C760R mutants and mRNA expression deficits in G607S mutants.

**Electronic supplementary material:**

The online version of this article (10.1186/s40478-017-0486-9) contains supplementary material, which is available to authorized users.

## Introduction

Distal arthrogryposis (DA) is a group of congenital movement disorders causing contracture phenotypes mainly in distal joints of patients’ limbs [1]. In addition to the severe phenotypes in distal limb joints, DA also affects the movement of proximal limb joints and other body parts from birth onward, and can be divided into at least 10 different subtypes depending on the affected regions [1]. In most cases, the disease is autosomal dominantly inherited and the patients often have a mutation in genes encoding the muscle contraction apparatus. Endothelin-converting enzyme-like 1 (*ECEL1*, also termed *DINE* in rodents [13, 16]), a membrane-bound metalloprotease, has recently been identified as a gene responsible for the autosomal recessive type 5 form of DA (DA5) [MIM 615065] [8, 21], originally characterized by its ocular phenotypes [10]. In contrast to other causal genes of DA, ECEL1 is predominantly expressed in fetal and adult motor neurons [8, 34], suggesting a unique neurogenic pathogenesis in patients with *ECEL1* mutations. In addition to limb contractures, patients with *ECEL1* mutations have ptosis and strabismus with variable expressivity and penetrance. Based on the ophthalmic abnormality, previous clinical studies have pointed out that *ECEL1* could be considered as a causal gene of another congenital contracture disorder termed congenital cranial dysinnervation disorder (CCDD) [14, 30]. This is a heterogeneous group of syndromes resulting from aberrant wiring of motor nerves in the head muscles [6, 26], and not from malformations of the eye itself. Further, some patients with *ECEL1* mutations have been shown to develop a restrictive pulmonary insufficiency [8].

**Fig. 1:**
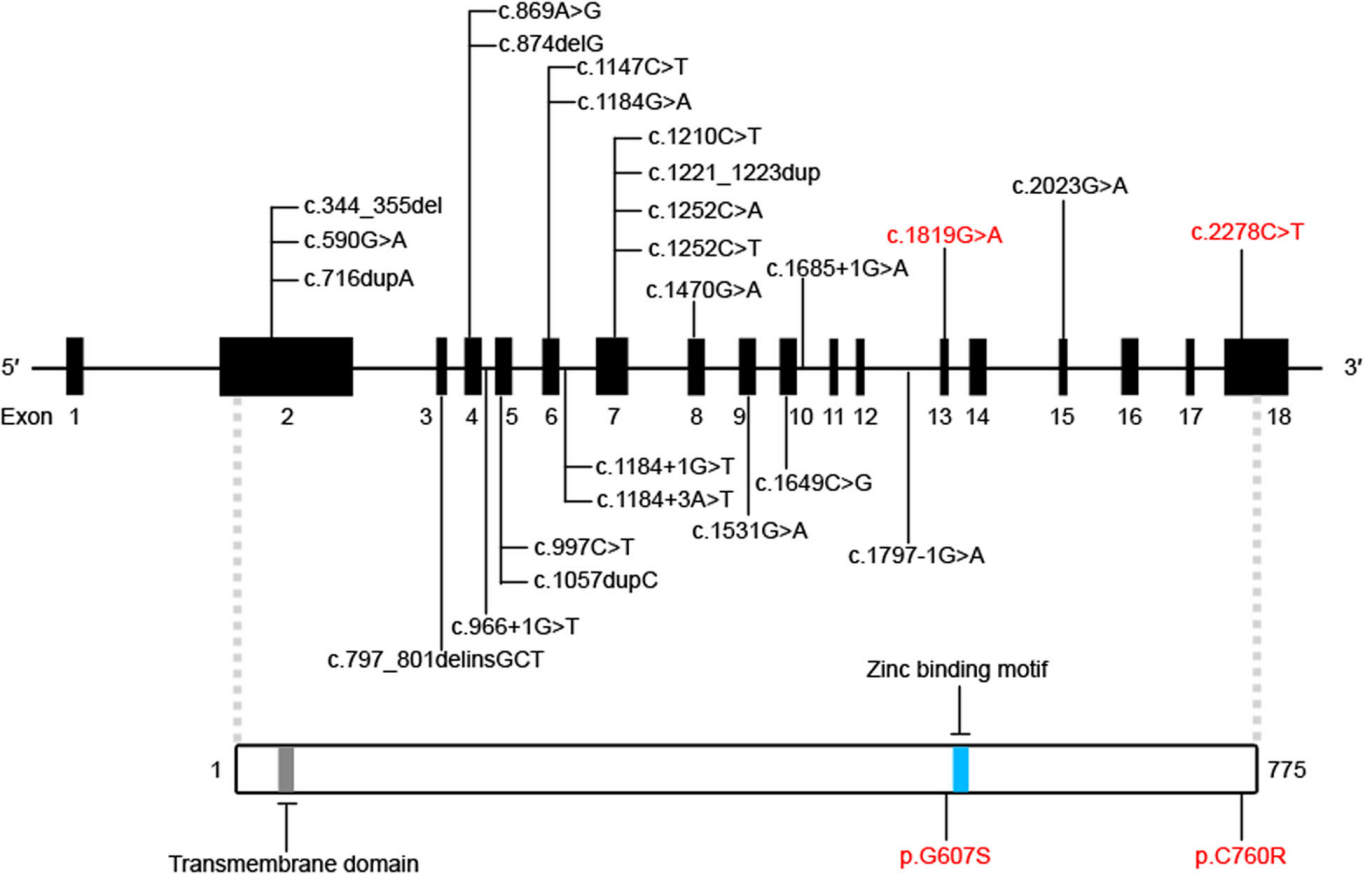
Previously reported *ECEL1* mutations. All reported pathogenic mutations are presented in the *ECEL1* genomic structure (upper panel). The 18 exons of *ECEL1* are shown in black boxes. The corresponding mutations that we introduced into our mouse models are shown in red. The consequences of the mutations at the protein level are indicated in the ECEL1 protein structure (lower panel). ECEL1 protein is a 775 amino acid transmembrane protein with a short cytosolic region and a long extracellular region. The zinc binding motif is expected to play a crucial role in the enzymatic activity

With the rapid progress of genetic analyses using human material, 40 patients with *ECEL1* mutations have been classified into DA or CCDD (Fig. 1) [2, 3, 8, 9, 11, 21, 27, 30, 31]. Nonetheless, there remain significant challenges to uncovering the pathogenic mechanisms as well as the genotype-phenotype relationships of congenital contracture disorders for the following reasons. First, given that 30 out of 40 patients belong to consanguineous families, there is a possibility that the different genetic loads among the patients affects the clinical expressivities, as pointed out in recent whole exome sequencing analyses of 52 arthrogryposis patients [3]. Second, difficulty in obtaining biopsy samples hinders exploration of the molecular etiology of the pathogenic DA mutations. Third, in all cases, the number of the patients with a particular mutation is small. Given that there are large phenotypic variations in inter-familial and even in intra-familial DA patients [15], it is hard to find exact genotype-phenotype relationships by only clinical evaluation of such a small number of patients. To complement the above-mentioned intrinsic drawbacks in human clinical research, further experimental validation is needed to address the etiology as well as the genotype-phenotype relationships of *ECEL1* mutations.

The highly conserved amino acid sequences of ECEL1/DINE between humans and rodents and the dominant expression of ECEL1/DINE in neuronal tissues of both species [22, 34] make it possible to utilize mouse models to gain a basic understanding of the etiology of *ECEL1* mutations that cause DA. Previous gene disruption studies showed that *DINE*-deficient mice die immediately after birth due to respiratory failure [23, 29]. Morphological analyses at embryonic stages revealed that disruption of *DINE* leads to abnormal axonal arborization of phrenic motor nerves, which innervate the diaphragm muscle and play a crucial role in respiratory control [23]. More recently, systematic examination of motor innervation in more than 10 hindlimb muscles revealed that *DINE* deficiency also affected the axonal arborization of motor nerves, especially in foot muscles, partially consistent with the severe contracture phenotype seen in the distal limb joints of DA patients [24]. Importantly, *DINE* knock-in mice with a pathogenic missense mutation (p.C760R) identified in DA patients (Fig. 1) [8], reproduced the insufficient arborization of motor nerves in diaphragm muscle and limb muscles [24].

Previous mutant mouse studies clearly suggested that axonal arborization defects of motor nerves in respiratory muscle and limb muscles could at least partially explain the pathogenesis of respiratory failure and limb contractures in *ECEL1*-mutated DA, although there is some discordance in the severity of respiratory failure between the patients and the mouse models. However, it remains unclear whether patients with different *ECEL1* mutations develop their symptoms by a common pathogenic mechanism. Further, it raises the question of how each identified mutation affects ECEL1/DINE function. The present study aimed to understand the etiology of *ECEL1*-mutated congenital contracture disorders in more detail by using two distinct, relevant knock-in mouse lines with different pathogenic missense mutations.

## Materials and methods

### Animals

In addition to *DINE*-deficient mice [23] and C760R knock-in mice [24], newly developed G607S and C760G knock-in mice were used in this study (see below for detailed information). Homozygous mutant mice were obtained by intercrossing heterozygous parents. All mutant mice were crossbred with Hb9::eGFP mice [35] to label embryonic motor nerves with GFP. Noon on the day of vaginal plug detection was considered embryonic day 0.5 (E0.5). The genotype of *DINE*-deficient mice, Hb9::eGFP mice and C760R knock-in mice was determined using allele-specific primers as previously described [24].

### Generation of *DINE* knock-in mice with a missense mutation

*DINE* knock-in mice with a pathogenic mutation (G607S: replacement of Gly607 with Ser) were generated using the CRISPR/Cas9 system. The plasmid vector pX330 was a gift from Feng Zhang (Addgene plasmid # 42230) [7], expressing single-guide RNA (sgRNA), as well as Cas9. The CRISPR Design Tool (http://tools.genome-engineering.org) was used to design sgRNA in silico. The target sequence and protospacer adjacent motif (PAM) sequence was TCTCTGAACTACGGGGGTAT**TGG (**The PAM sequence is shown in bold letters). The sgRNA and Cas9 mRNA were synthesized in vitro using commercially available kits as previously described [24]. The sgRNA (10 ng/μl) and Cas9 mRNA (10 ng/μl) were injected into C57BL/6 mouse zygotes in the presence of 90 bp single-stranded oligonucleotides (ssODN) (10 ng/μl) using a microinjection system under standard conditions. The ssODN sequence was: CAGTCGTCATAGCCATGGGTCAGTTCGTGCCCAATGATGGTGCtAATACCCCCGTAGTTCAGAGACCTGGGCCCACAGCAGCAGGTTAAA (the mutation site is shown as a lowercase letter). The zygotes were cultured in culture medium at 37 °C in 5% CO_2_ up to the two-cell embryo stage and then the embryos were transferred into the oviducts of recipient mice. Tail genomic DNA was amplified using a specific primer set: forward 5′-ACTATCCGTCCTTCCCCTCC-3′ and reverse 5′-AGATCTCTGGGGCCTCTCTG-3′. The PCR product was used to confirm the mouse genotype by sequencing with the forward primer or restriction fragment length polymorphism analyses with Ban I.

Similar methods were used for generation of another *DINE* knock-in mouse with an artificial mutation (C760G: replacement of Cys760 with Gly) with appropriate modification of the target sequence as well as the ssODN sequence: the target and PAM sequences were CCCAGTTTGAGGAATTCGGC**CGG**. The ssODN sequence was: CACCCCAGGGTCCTGGGCAGCGTATCCCAGTTTGAGGAATTCGGCCGaGCCTTCCACgGTCCCAAGGACTCTCCCATGAACCCCGTCCAT. Two mutation sites are shown as lowercase letters and the first mutation (a) is a synonymous mutation to inhibit re-cutting by Cas9.

### Off-target effect analysis

We chose five potential off-target sites (OT1-OT5) of the target sequence for G607S knock-in generation (TCTCTGAACTACGGGGGTAT**TGG)** using the CRISPR design tool (http://tools.genome-engineering.org): OT1 TCTaaGAACTACtGGGGTAT**GGG**, OT2 agTgTGAACTACGGGGGTAa**CAG**, OT3 TCTtTGAACcACGGGGGgAT**GAG**, OT4 TCTaaGtACTACaGGGGTAT**AAG**, OT5 aCaCTGAAgTACaGGGGTAT**GAG**. Mutation sites and PAM sequences are shown as lowercase letters and bold letters, respectively. The surveyor assay was performed to detect the CRISPR/Cas9-induced mutations, as previously described [24]. Target fragments on the off-target sites were amplified using specific primer sets: OT1 forward 5′-TGTTAACAAAATGGAAATGATTCAA-3′ and OT1 reverse 5′-TCAGAGTTCCATGTGGCAGTA-3′, OT2 forward 5′-TCCTTCTCAGATCCCTTGTCA-3′ and OT2 reverse 5′-TGCCATGGATGTAAATCATCA-3′, OT3 forward 5′-CGGTGGGTGGTGTTTCTTAT-3′ and OT3 reverse 5′-GGTGGCAGGAGTTCCTTCTT-3′, OT4 forward 5′-GGCTGCAGGCAGGTAGTTCT-3′ and OT4 reverse 5′-TCCCAAACAGTTAATGAATCAGTG-3′, OT5 forward 5′-TTCTTCTGGAGTCCCCAATG-3′ and OT5 5′-reverse 5′- GCACAGGTTTTTGGAGGAAA-3′.

### Immunohistochemistry

E12.5 mouse embryos were collected, fixed in 4% paraformaldehyde (PFA) at 4 °C for 2 h, then immersed in PBS containing 30% sucrose for two additional days. The tissue samples were embedded in optimal cutting temperature (OCT) compound (Sakura Finetek, Torrance, CA, USA), and stored at −80 °C until use. Serial 20 μm sections were cut using a cryostat microtome. Immunohistochemistry was performed as described previously [17], with minor alternations. Briefly, the sections were rinsed three times in PBS, permeabilized by immersion in absolute methanol for 6 min at −30 °C, rinsed in PBS for 30 min, and blocked for 30 min in 0.3% Triton X-100 and 0.2% bovine serum albumin (BSA) in PBS. The sections were then incubated with goat anti-DINE primary antibody (1:500; Santa Cruz Biotechnology, Dallas, TX, USA) in the blocking solution at room temperature overnight. After washing, anti-goat secondary antibody conjugated with Alexa Fluor 546 (1:500; Invitrogen, Carlsbad, CA, USA) was applied for 50 min and then tissues were rinsed three times in PBS. The sections were visualized using confocal microscopy (FV1200; Olympus, Tokyo, Japan).

### Whole-mount immunohistochemistry

Whole-mount immunohistochemistry was performed as previously described [24]. Embryonic tissues were incubated with rabbit anti-GFP primary antibody (1:500; Life Technologies, Carlsbad, CA, USA) to detect the motor nerves. For detection of cranial nerves, embryonic heads were incubated with mouse monoclonal antibody 2H3 (1:500; Developmental Studies Hybridoma Bank, University of Iowa, IA, USA). Subsequently, tissues samples were incubated with Alexa Fluor 488 goat anti-rabbit secondary antibody (1:500; Invitrogen) and Alexa Fluor 546 goat anti-mouse secondary antibody (1:500; Invitrogen). After PBS washes, tissue samples were dehydrated through a methanol series (30%, 50%, 80%, and 100% for 30 min each), cleared with benzyl alcohol-benzyl benzoate (BABB), and imaged using confocal microscopy FV1200 (Olympus).

### Image analysis

An FV1200 laser-scanning confocal microscope (Olympus) was used to acquire the confocal images. We used 10x and 20x dry objective lenses to visualize motor nerves. Multiple adjacent regions of embryonic mouse limb, head, or individual muscles were imaged using a motorized xy stage module. Image analyses were performed using IMARIS software (Bitplane, Zurich, Switzerland). To evaluate the extent of motor innervation in each individual skeletal muscle, all stacked images were converted into 3D images and the motor nerve terminals were semiautomatically traced using the filament tracer function. The number of terminal points was automatically calculated. The measurement of the nerve length of ocular motor nerves was performed using the measurement point function. To appropriately compare results between samples, all data were processed using the same criteria.

### RT-PCR

Total RNA was isolated from embryonic spinal cords using the RNeasy mini kit (Qiagen, Valencia, CA, USA) according to the manufacturer’s protocol. For quantification of DINE mRNA expression, total RNA (1 μg aliquots) was converted to cDNA by ReverTra Ace (Toyobo, Osaka, Japan) and an oligo (dT) primer. The resultant cDNA was diluted 1:50 with distilled water. The quantitative PCR (qPCR) procedures were performed as previously described [24]. TaqMan Gene Expression Assays (Applied Biosystems, Waltham, MA, USA) for mouse actin beta (ACTB) (Mm00607939_s1) and ECEL1 (Mm00469610_m1) were used for specific target amplification. Relative mRNA expression was calculated by using the comparative cycle threshold (CT) method, and then normalized to endogenous ACTB mRNA expression for each sample. The CT value was obtained from the amplification plot with the aid of SDS software (Applied Biosystems).

For qualitative analysis of the mutant DINE transcript, total RNA (1 μg aliquots) was converted to cDNA using ReverTra Ace (Toyobo, Osaka, Japan) with random primers. The cDNA was amplified using the following specific primers: forward 5′-CCACCCTGTATGACCCAGAC-3′, reverse 5′-ATAGAGGCGAACGATGCACT-3′.

### Preparation of membrane fractions from embryonic mouse spinal cord

E17.5 mice spinal cords were homogenized in 50 mM Tris-HCl (pH 7.4) containing protease inhibitor cocktail complete mini (Roche Diagnostics, Indianapolis, IN, USA) (homogenizing buffer). After one centrifugation, the supernatant was centrifuged at 20,000 g for 30 min at 4 °C. The pellet fraction was collected and then solubilized with homogenizing buffer containing 1% Triton X-100 for 30 min at 4 °C and centrifuged again at 20,000 g for 30 min at 4 °C. The supernatants served as protein samples for further analyses.

### Glycosidase treatment

Protein samples (100 μg) were boiled in Glycoprotein Denaturing Buffer (NEB, Ipswich, MA, USA) for 10 min. For Endoglycosidase H (Endo H) treatment, samples were added to a reaction containing GlycoBuffer 3 (NEB) and endo H (1000 units, NEB) and incubated at 37 °C for 1 h. For PNGase F treatment, samples were added to a reaction containing GlycoBuffer 2 (NEB), 1% Nonidet P-40 (NEB), and PNGase F (1000 units, NEB) and incubated at 37 °C for 1 h.

### Western blotting analysis

Protein samples (50 μg) with or without glycosidase treatment were separated by 5–20% gradient sodium dodecyl sulfate polyacrylamide gel electrophoresis (SDS-PAGE), followed by transfer to polyvinylidene fluoride (PVDF) membranes. After incubating with 2% enhanced chemiluminescence (ECL) blocking reagent (GE Healthcare, Buckinghamshire, UK), the membranes were incubated with goat anti-DINE antibody (1:500; Santa Cruz Biotechnology) at 4 °C overnight. The membranes were repeatedly washed then incubated with horseradish peroxidase-conjugated anti-goat IgG secondary antibody (1:5000; Vector, Burlingame, CA, USA). Anti-GAPDH antibody (1:5000; Trevigen, Gaithersburg, MD, USA) was used for the control experiments. Each set of experiments was repeated at least three times to confirm results.

### Statistical analyses

Data were first analyzed for normal distribution and equal variance. When normally distributed, two independent samples were statistically analyzed using a two-tailed Student’s *t* test or Welch’s *t* test. If the data did not pass normality testing, the Mann-Whitney *U* test was used. For three independent samples, the data was statistically analyzed using one-way ANOVA for normal distributions or the Kruskal-Wallis test followed by the Steel-Dwass test for non-normal distributions, with *p* < 0.05 considered significant. All analyses were completed with Statcel 3 (add-in software for Excel, Microsoft, USA).

## Results

### Generation of *DINE* knock-in mice with a pathogenic mutation (G607S)

**Fig. 2:**
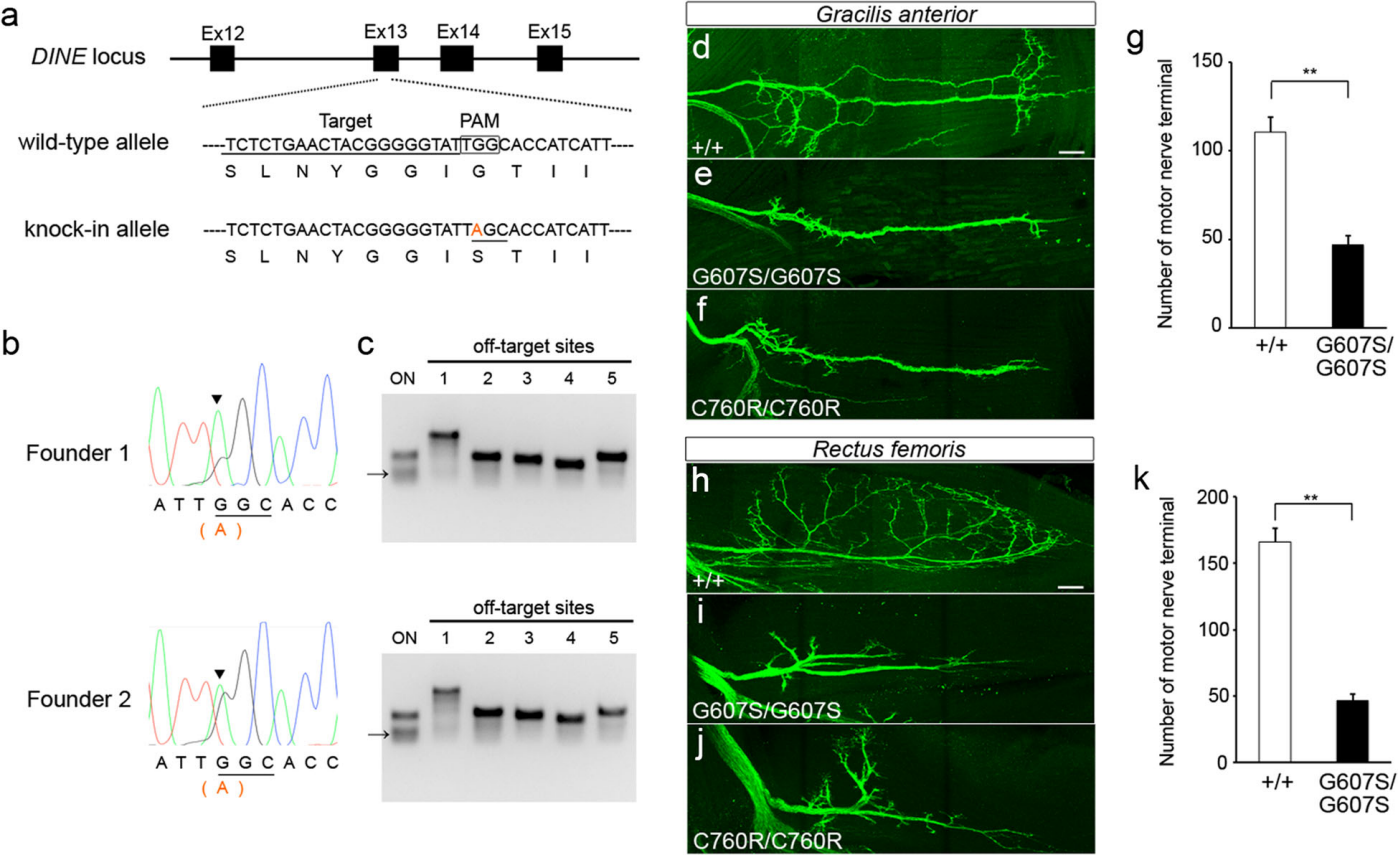
Generation of *DINE* G607S knock-in mice using CRISPR/Cas9. **a** The original sequence in the *DINE* locus was changed into a knock-in allele containing a pathogenic missense mutation (G607S). Four exons (Ex12–15) are shown in black boxes. The sgRNA target sequence and PAM sequence are marked with an underline and a square, respectively. The mutated nucleotide is shown in orange in the knock-in allele. The amino acid sequence of the original DINE protein and that of the mutated protein are juxtaposed with the base sequences in the wild-type allele and the knock-in allele, respectively. **b** Electropherograms showing DNA sequencing results from two G607S founder mice (Founder 1 and Founder 2). The intact peak (**g**) and mutated peak (**a**) can be observed at the mutated site (shown by the black arrow head). **c** Off-target analyses with the mismatch-specific endonuclease were performed on five potential sites (OT1-OT5) using PCR products from the founder tail genomic DNA. Cleaved bands were specifically detected at the on-target site in the founder mice, whereas no cleaved bands were detected on any potential off-target sites. **d**–**k** Motor nerves were visualized by GFP immunoreactivity to compare the innervation pattern between E17.5 wild-type and homozygous mutant hindlimb muscles. The number of motor nerve terminals was significantly reduced in G607S mutant gracilis anterior (**d**–**g**) and rectus femoris (**h**–**k**) muscles. Mean ± SEM, two-tailed Student’s *t* test, **p < 0.01, *n* = 5. Scale bar: 100 μm (**d**–**f**, **h**–**j**)

We previously generated a *DINE* knock-in mouse line carrying C760R as a relevant model of *ECEL1*-mutated DA, and detected axonal arborization defects of motor nerves in homozygous C760R mutant limb muscles [24]. However, given that the observed expressivities vary in some affected regions in patients with *ECEL1* mutations [2, 30], phenotypic comparison with another knock-in mouse with a distinct pathogenic mutation is necessitated to judge whether axonal arborization defects are a common mechanism in the pathogenesis of *ECEL1*-mutated DA. Notably, Shaaban et al. have reported two siblings with a missense c.1819G > A mutation (p.G607S) (Fig. 1) in the *ECEL1* gene that presented with significant ophthalmoplegia and less pronounced contractures in the distal joints of lower limbs [30]. Because the symptoms did not meet the major criteria for the diagnosis of DA, the authors concluded that the two siblings differed from other patients with different *ECEL1* pathogenic mutations. To experimentally compare the pathogenic effects between the C760R and G607S mutations, we have generated a *DINE* knock-in mouse line carrying G607S using the CRISPR/Cas9 system. We designed a target sequence of sgRNA in the region close to the mutation site, as well as a 90 bp single-stranded DNA (ssDNA) with the pathogenic mutation as the DNA template (Fig. 2a). The CRISPR/Cas9 tools were injected into 200 mouse zygotes and then 158 normally developed two-cell embryos were transferred into recipient female mice. A total of 71 mice were born normally. We performed sequencing analyses using the PCR amplified target region to verify the genotype of the CRISPR-injected mice and successfully obtained 7 F0 mutant mice. We selected two male mice (Founder 1 and Founder 2) with a dominant mutated peak in electropherograms (Fig. 2b) and used these founder mice for expansion of the mouse colony. Off-target analyses using a mismatch cleavage enzyme showed no off-target mutations at five potential sites in the founders (Fig. 2c). The results were also confirmed using direct sequencing analyses (data not shown). After confirmation of the transmission of the desired mutation from the founders to their offspring, we generated G607S mutant mice with motor neuron-specific expression of GFP by crossing with Hb9::eGFP mice [35].

### Axonal arborization defects of motor nerves in G607S mutant hindlimb muscles

**Fig. 3:**
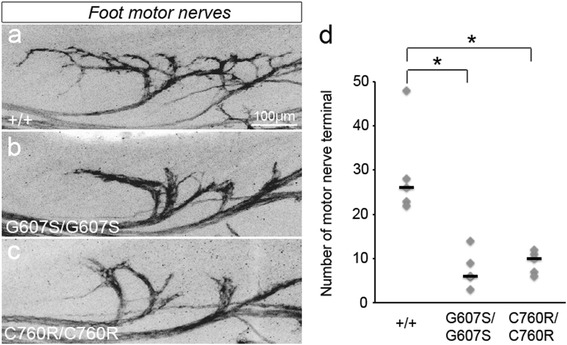
Axonal arborization defects in G607S hindlimb and foot muscles. Motor nerves were visualized by GFP immunoreactivity to compare the innervation pattern between E17.5 wild-type (**a**) and homozygous knock-in mutant (**b**, **c**) foot muscles. The terminal number of motor nerves is individually plotted. Black bars represent the median. The number of motor nerve terminals was significantly reduced in both G607S and C760R mutant muscles (**d**). Kruskal-Wallis test followed by the Steel-Dwass test, *p < 0.05. *n* = 5. Scale bar: 100 μm (**a**–**c**)

First, we performed whole-mount immunostaining with an anti-GFP antibody in the homozygous G607S mutant diaphragm. Identical to the *DINE*-deficient [23] and C760R diaphragm [24], homozygous G607S mutant mice displayed impaired axonal arborization of motor nerves in the diaphragm, possibly leading to perinatal lethality (Additional file 1: Figure S1). In fact, we never obtained any homozygous G607S mutant pups by mating heterozygous mutants. Next, we performed the same morphological analyses in hindlimb muscles at E17.5 to detect the morphology of embryonic motor nerves and to compare the morphological phenotypes of motor innervation between C760R and G607S mutant mice. Compared with wild-type hindlimbs, abnormal motor innervation was detected in a number of G607S mutant hindlimb muscles (data not shown). In order to quantify the number of motor nerve terminals, we selected two hindlimb muscles, the gracilis anterior muscle and the rectus femoris muscle, which are severely affected in *DINE*-deficient embryos and C760R mutant embryos [24]. The number of motor nerve terminals was clearly decreased in the G607S mutant gracilis anterior muscles (46.8 ± 5.0 in G607S mutant embryos, *n* = 5, vs. 110.6 ± 8.4 in wild-type embryos, *n* = 5; *p* = 0.0002, two-tailed Student’s *t* test) (Fig. 2d–g), as well as in the rectus femoris muscle (46.4 ± 4.9 in G607S mutant embryos, *n* = 5, vs. 166.4 ± 9.5 in wild-type embryos, *n* = 5; *p* = 0.000003, two-tailed Student’s *t* test) (Fig. 2h–k), similar to the C760R mutant muscles. We also performed the same morphological analyses in foot muscles in order to directly compare the innervation of motor nerves between G607S and C760R mutants. In contrast to the phenotypic discordance in foot muscles between patients with G607S [30] and DA patients with C760R [8], G607S mutant mice displayed the same clear axonal arborization defects in the foot muscle as the C760R mutant (median terminal number 26 in wild-type embryos, *n* = 5; 6 in G607S mutant embryos, *n* = 5; 10 in C760R mutant embryos, *n* = 5; Kruskal-Wallis test followed by the Steel-Dwass test, *p* < 0.05) (Fig. 3a–d). These results suggested that G607S and C760R mutations similarly affected intramuscular axonal arborization of motor nerves in the hindlimbs.

### Axon guidance defects in abducens nerves in *DINE* mutant mice

**Fig. 4:**
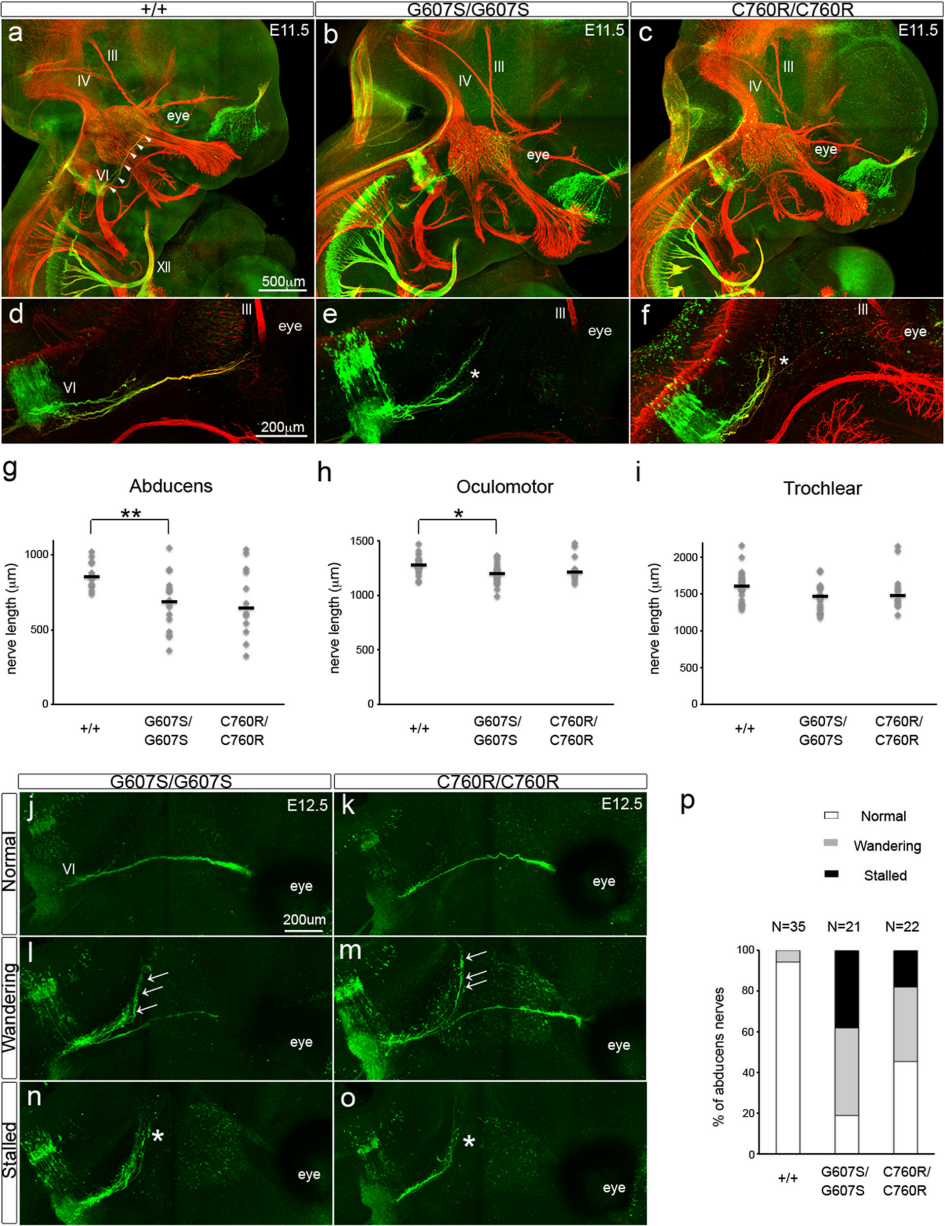
Axon guidance defects in *DINE* mutant abducens nerves. Whole-mount fluorescent immunostaining with anti-neurofilament (red) and anti-GFP (green) antibodies of E11.5 wild-type (**a**), homozygous G607S mutant (**b**), and homozygous C760R mutant (**c**) embryos. White arrowheads represent the pathway of wild-type abducens nerves. **d**–**f** High-magnification views of E11.5 abducens nerves. The axon terminal (*) of mutant abducens nerves (**e**, **f**). **g** Quantification of nerve length of abducens, (**h**) oculomotor, and (**i**) trochlear nerves. Kruskal-Wallis test followed by the Steel-Dwass test for the abducens and oculomotor nerves, one-way ANOVA for the trochlear nerve, *p < 0.05, **p < 0.01. **j**–**o** The three axon guidance patterns in E12.5 homozygous mutant abducens nerves. Substantial numbers of wandering nerves (arrows) were present in homozygous mutant abducens nerves (**l**, **m**). The wandering phenotype was defined as abducens nerves with inappropriately extending nerves of substantial length (> 30% of appropriately innervated nerves). The edge (*) of mutant abducens nerves was far from the target area (**n**, **o**). The stalled phenotype was defined as abducens nerves not reaching the target area. **p** The ratio of abducens nerves with abnormal axon guidance. Scale bar: 500 μm (a–c), 200 μm (**d**–**f**, **j**–**o**). Abbreviations: III, oculomotor nerve; IV, trochlear nerve; VI, abducens nerve; XII, hypoglossal nerve

Next, we explored whether abnormal innervation also occurred in the mutant cranial motor nerves. Given that the ocular phenotype is the most prominent feature of *ECEL1*-related DA [14] as well as the patients with G607S [30], it is important to assess the function of DINE in cranial motor nerves, especially ocular motor nerves. To this end, whole-mount immunostaining with anti-GFP antibody and anti-neurofilament antibody was performed on embryonic head samples. Among the three ocular motor nerves (oculomotor, trochlear and abducens nerves) innervating extraocular muscles, only the abducens nerves were fluorescently labeled in Hb9::eGFP mice (Fig. 4a–c), consistent with previous results [12]. Both oculomotor nerves and trochlear nerves were visualized only with anti-neurofilament antibody (Fig. 4a–c). At E11.5, all wild-type ocular motor nerves correctly chose their pathway toward the target muscles around the eye (Fig. 4a, d). In contrast, the abducens nerves stopped extending their axons toward the target muscles in a number of homozygous G607S mutant embryos (Fig. 4b, e). The oculomotor nerves and trochlear nerves showed a normal trajectory to the target muscles in these mutant embryos (Fig. 4b, e). Interestingly, the axon guidance defects of the abducens nerves were similarly observed in homozygous C760R mutants (Fig. 4c, f). Subsequent quantitative analyses revealed that a drastic reduction of nerve length could be detected in a substantial number of homozygous mutant abducens nerves (median nerve length 855.4 μm in wild-type embryos, *n* = 21; 685.2 μm in G607S mutant embryos, *n* = 16; 642.4 μm in C760R mutant embryos, *n* = 12; Kruskal-Wallis test followed by the Steel-Dwass test, wild-type vs. G607S mutant, *p* < 0.01) (Fig. 4g). In contrast, such a reduction was not observed in the homozygous mutant oculomotor nerve, although the difference in nerve length between wild-types and the homozygous G607S mutants reached statistical significance (median nerve length 1280 μm in wild-type embryos, *n* = 22; 1216 μm in G607S mutant embryos, *n* = 20; 1198 μm in C760R mutant embryos, *n* = 12; Kruskal-Wallis test followed by the Steel-Dwass test, wild-type vs. G607S mutant, *p* < 0.05) (Fig. 4h). The length of the trochlear nerve was not significantly different between wild-type and homozygous mutant embryos (median nerve length 1607 μm in wild-type embryos, *n* = 22; 1479 μm in G607S mutant embryos, *n* = 20; 1469 μm in C760R mutant embryos, *n* = 12; one-way ANOVA, *p* > 0.05) (Fig. 4i). To exclude the possibility that the observed phenotype in some mutant embryos was just due to developmental delay, we performed the same morphological analyses in abducens nerves at a later stage. At E12.5, all wild-type abducens nerves reached their target muscles. While some of the homozygous G607S mutant abducens nerves normally innervated the extraocular muscles (Fig. 4j), substantial numbers of abducens nerves followed the wrong pathway in some homozygous mutant embryos (‘wandering’ phenotype; Fig. 4l). In addition, a number of mutant abducens nerves stopped extending their axons toward their target muscles without any correct innervation (‘stalled’ phenotype; Fig. 4n). These axon guidance defects were also observed in homozygous C760R mutant embryos with variable penetrance (Fig. 4k–o). Quantitative analyses with a substantial number of embryos (*n* = 78) clearly revealed that abnormal axon guidance could be detected in over half of the homozygous mutants (Fig. 4p). These results indicated that both G607S and C760R mutations frequently led to axon guidance defects in abducens nerves.

### Drastic reduction of DINE expression in G607S mutant spinal cord

**Fig. 5:**
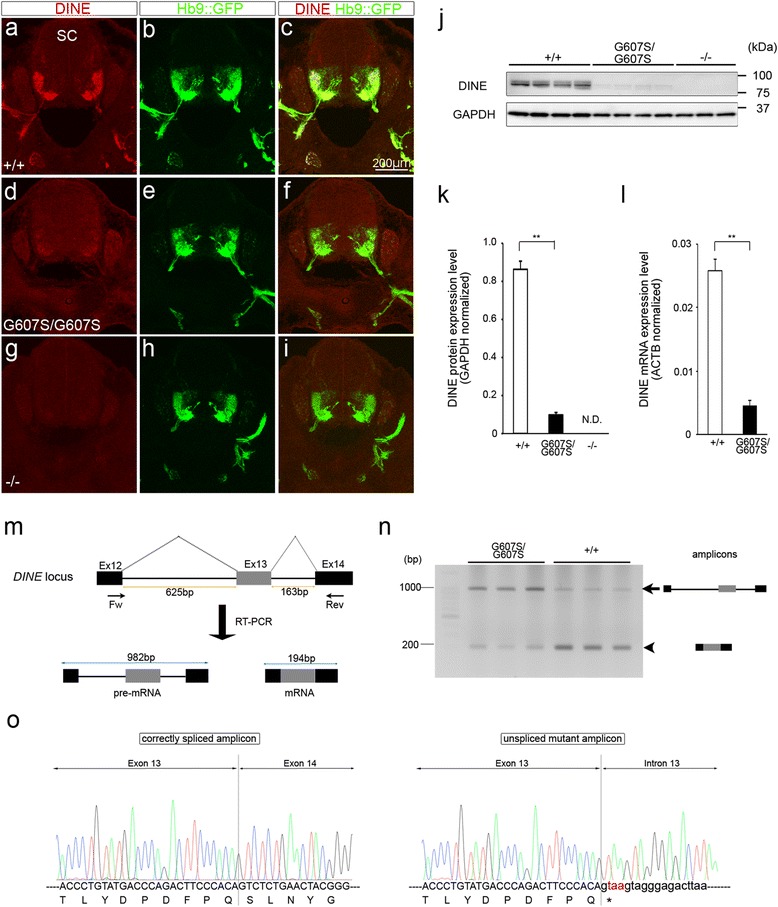
Expression analyses of DINE in G607S mutant spinal cords. Immunohistochemical analysis with anti-DINE antibody in horizontal sections of E12.5 mouse spinal cords from wild-type (**a**–**c**), homozygous G607S mutant (**d**–**f**) and *DINE*-deficient embryos (**g**–**i**). **j** Western blotting analysis using spinal cord protein from wild-type, homozygous G607S mutant, and *DINE*-deficient embryos. **k** Quantitative evaluation of the western blotting. Two-tailed Student’s *t* test, **p < 0.01. **l** Quantitative expression analysis using spinal cord mRNA from wild-type and homozygous G607S mutant embryos. Two-tailed Student’s *t* test, **p < 0.01. **m** Schematic image of qualitative RT-PCR analysis for the mutant transcript. **n** Wild-type and mutant transcripts were evaluated by RT-PCR. The arrow and arrowhead indicate the size of the pre-mRNA and mRNA products, respectively. **o** Electropherograms showing the sequencing results of RT-PCR products from wild-type (left) and homozygous G607S mutant (right). Scale bar: 200 μm (**a**–**i**). SC, spinal cord

In contrast to nonsense mutations, which lead to loss of full-length protein, the biological interpretation of a missense mutation with only one amino acid exchange is often challenging. In order to characterize the consequences of G607S on DINE function, we first performed immunohistochemical analyses of G607S mutant embryonic spinal cords with an anti-DINE antibody. In contrast to wild-type samples (Fig. 5a–c), only a faint positive signal could be detected in homozygous G607S mutant spinal cords (Fig. 5d–f), similar to *DINE*-deficient spinal cords (Fig. 5g-i). The immunohistochemical data was subsequently confirmed using western blotting analysis. DINE protein expression levels were significantly decreased in homozygous mutant spinal cords (0.098 ± 0.012 in G607S mutant embryos, *n* = 4 vs. 0.87 ± 0.039 in wild-type embryos, *n* = 4; *p* = 0.000002, two-tailed Student’s *t* test) (Fig. 5j, k). We hypothesized that the missense mutation could lead to reduced protein expression via the response against formation of misfolded proteins. To validate the possibility, we further performed quantitative real time PCR in order to examine whether DINE expression was maintained at a transcriptional level. Unexpectedly, the mRNA expression level was significantly reduced in homozygous mutant samples compared with wild-type samples, similar to the protein level (0.003 ± 0.001 in *DINE*-deficient embryos, *n* = 3 vs. 0.023 ± 0.002 in wild-type embryos, *n* = 3; *p* = 0.002, two-tailed Student’s *t* test) (Fig. 5l). To further explore the mechanism underlying the reduced mRNA expression, DINE transcripts containing exon 13 with the pathogenic mutation site were reverse transcribed and then amplified using a forward primer on exon 12 and a reverse primer on exon 13 (Fig. 5m). The mature mRNA product was clearly observed with a faint pre-mRNA product in wild-type samples (Fig. 5n). In contrast, in homozygous mutant samples, a strong pre-mRNA product could be detected in addition to the mature mRNA, which was of negligible intensity (Fig. 5n). We also detected a similar tendency in heterozygous adult G607S mutant hypothalamus (Additional file 2: Figure S2), although the band intensity of the pre-mRNA product was much weaker than that in the homozygous mutant spinal cords. We subsequently performed sequencing analyses on the TA cloned RT-PCR products and confirmed that the homozygous mutant transcript contained additional sequences of intron 12 and intron 13 (Fig. 5o), indicating that splicing defects occurred in the mutant samples. Importantly, due to the second to fourth nucleotide sequences of intron 12, the aberrant transcript with intron retention contained TAA, a nucleotide sequence corresponding to a translation termination codon, on the reading frame (Fig. 5o). As it is widely accepted that an in-frame premature translation termination codon induces an mRNA degradation pathway termed nonsense-mediated decay [18], these results provide the first evidence that splicing defect-induced mRNA degradation could be the primary functional consequence of the G607S mutation.

### Axonal transport of C760R DINE protein is impaired in spinal cord

**Fig. 6:**
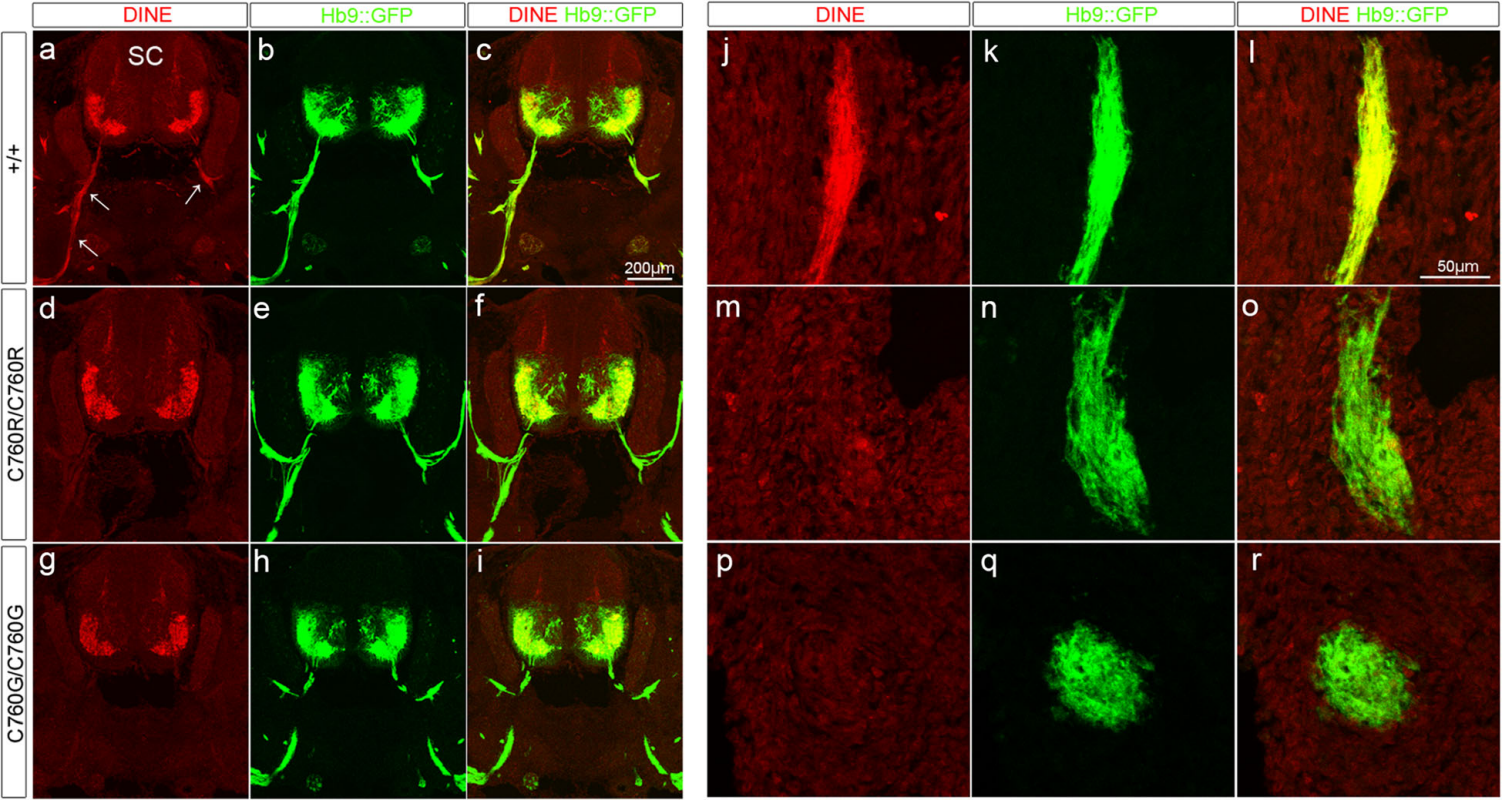
Altered localization of C760R mutant protein. Immunohistochemical analyses with anti-DINE antibody in horizontal sections of E12.5 mouse spinal cords (**a**–**i**) and diaphragm muscles (**j**–**r**). In the case of wild-type spinal cord, DINE immunoreactivity was detected in both motor neuron soma and axons (arrows), which were labeled with GFP (**a**–**c**). Similar immunoreactivity could be detected at the end of the phrenic motor nerves innervating diaphragm muscle (**j**–**l**). In contrast, DINE expression was drastically decreased in C760R (**d**–**f**, **m**–**o**) as well as C760G motor axons (**g**–**i**, **p**–**r**)

Finally, we explored the functional consequence of the pathogenic C760R missense mutation. Given that the homologous residue to cysteine 760 in the DINE protein has been shown to form a disulfide bond in a family member protein [19], we hypothesized that this residue plays crucial roles in the appropriate protein conformation of DINE. To test the possibility, we first examined whether the posttranslational modifications of mutant DINE protein had occurred appropriately. Previous cell culture experiments showed that most DINE protein is Endoglycosidase H (endo H)-sensitive and retained in the endoplasmic reticulum (ER) but some DINE protein is endo H-resistant and plasma membrane-associated [4]. Consistent with the previous cell culture study, we could discriminate some endo H-resistant DINE protein from the endo H-sensitive in wild-type spinal cords (Additional file 3: Figure S3). In contrast, all mutant DINE protein was endo H-sensitive (Additional file 3: Figure S3). These results suggested that the mutant DINE protein was not appropriately glycosylated, which may result in abnormal protein conformation. We could not detect any different patterns in samples digested with another glycosidase PNGase F, which cleaves glycosylation added in both the ER and the golgi complex (Additional file 3: Figure S3). Next, we performed immunohistochemical analyses with an anti-DINE antibody to gain further information about the localization of the mutant DINE protein. In wild-type E12.5 embryos, DINE was expressed in motor neuron soma and axons in ventral spinal cords and all DINE positive signals were colocalized with GFP positive motor neurons (Fig. 6a–c). In contrast, DINE expression was drastically decreased in homozygous C760R mutant motor axons, which were clearly visualized with GFP signals, but not in the soma (Fig. 6d–f). We also explored whether mislocalization of the mutant protein occurred at distal parts of motor nerves. Whereas DINE expression was colocalized with GFP positive motor axons in wild-type diaphragm muscle (Fig. 6j–l), such positive immunoreactivity could not be detected in mutant diaphragm muscle (Fig. 6m–o).

The homologous residue in a family member protein to cysteine 760 in the DINE protein has been shown to form a disulfide bond [19], but there is no direct evidence showing that cysteine 760 of DINE actually forms a disulfide bond. Thus, it remains unclear whether the pathogenic effect of C760R results from the lack of a disulfide bond or an arginine-induced conformational change. To further discriminate the pathogenic possibilities, we generated a new *DINE* knock-in mouse with an artificial substitution of cysteine 760 with glycine, another small non-charged amino acid. This C760G mutant produced identical immunohistochemical results to the C760R mutant (Fig. 6g–i, p–r). These results indicate that cysteine 760 contributes to the appropriate conformation of DINE protein, possibly via a disulfide bond, and this conformational change possibly promotes the axonal transport of DINE.

## Discussion

In this study, we focused on two *ECEL1*/*DINE* missense mutations that were independently identified in patients with distinct congenital contracture disorders, and evaluated the functional consequences of each mutation using relevant knock-in mouse models. Morphological analyses of the newly generated G607S mutant mice revealed that the mutant embryos displayed reduced axonal arborization of motor nerves in hindlimb muscles, the same as C760R mutants. We also identified that a substantial number of G607S and C760R mutant abducens nerves displayed wandering or stalled phenotypes on the pathway toward the target muscles. Furthermore, biochemical and immunohistochemical analyses revealed that a drastic reduction of DINE mRNA levels occurred in G607S mutant spinal cords, whereas a lack of DINE protein was seen in C760R mutant spinal motor nerves. These results provide the first evidence that both G607S and C760R mutations in the *ECEL1/DINE* gene lead to the same clinically relevant phenotypes via discrete functional effects (Table 1).

**Table 1:** Information of three distinct *DINE* mutant mice

Name	Generation method	Mutation type	DINE mRNA level	DINE protein level	Lethality ^a^	Motor nerves in hindlimb muscles ^b^	Abducens motor nerves	Reference
*DINE*-deficient	gene targeting	gene disruption	complete loss	complete loss	Yes	axonal arborization defect	ND	[23, 24]
C760R knock-in	CRISPR/Cas9	missense mutation	almost same	altered localization	Yes	axonal arborization defect	axon guidance defects ^c^(stalled or wandering)	[24]
G607S knock-in	CRISPR/Cas9	missense mutation	strongly reduced (due to abnormal splicing)	strongly reduced	Yes	axonal arborization defect	axon guidance defects ^c^(stalled or wandering)	

^a^ Mutant mice die immediately after birth due to respiratory failure; ^b^ Hindlimb muscles means gracilis anterior, rectus femoris and foot muscles; ND, no data available; ^c^ The penetrance and expressivity were varied among samples

Although *ECEL1* was originally identified as a gene responsible for DA, a previous clinical study noted the presence of dominant ocular phenotypes in addition to the absence of hindlimb contracture phenotypes in patients with the *ECEL1* G607S mutation, resulting in another congenital contracture disorder termed CCDD. However, further experimental studies were needed to validate the genotype-phenotype relationship of the G607S mutation and CCDD, not only because the clinical study evaluated only two siblings with the mutation, but also because the phenotypic expressivity often differs among patients with *ECEL1* mutations. In this study, we utilized our two distinct knock-in mouse strains as two different congenital contracture disorder models (i.e. C760R for DA, G607S for CCDD), and compared morphological phenotypes of both cranial and spinal motor nerves. Consistent with the abnormal ocular phenotype observed in the patients with *ECEL1* mutations, our morphological analyses in embryonic head revealed that the two different mutant lines similarly affected axon guidance of abducens nerves. Notably, our mutant mice reproduced the variable expressivity as well as the low penetrance seen in patients with *ECEL1* mutations in a previous clinical study [14]. These data provide the first evidence that axon guidance defects of abducens nerves could be a primary cause of CCDD with *ECEL1* mutations, and supports the possibility that the overlapping phenotypes of the *ECEL1* mutation causing DA and that causing CCDD could be explained by abnormal motor innervation of ocular motor nerves.

Our previous study demonstrated that both *DINE*-deficient mice and homozygous C760R mutant mice showed axonal arborization defects of motor nerves in the diaphragm muscle and the limb muscles without affecting the axon guidance toward their target muscles [23, 24]. In contrast to the phenotypes seen in spinal motor neurons, two distinct axon guidance defects, i.e. stalled and wandering, were newly identified in homozygous mutant abducens nerves. Because no clear abnormal nerve trajectory phenotypes could be detected in the other ocular motor nerves, the axon guidance defects seemed to have specifically occurred in the mutant abducens nerves. A number of adhesion molecules and signaling molecules have been shown to play crucial roles in axon guidance, but it remains incompletely understood how axon guidance can be correctly achieved in each cranial and spinal motor nerve. Although it is likely that the axon guidance mechanisms in the abducens nerve are different from those of other ocular nerves, the exact molecular mechanisms remain to be elucidated. Recently, Nugent et al. reported that knock-in mice with an α2-chimerin gain-of-function missense mutation, identified in CCDD patients, showed a similar stalled phenotype in the abducens nerves, with variable penetrance [25]. More importantly, they also demonstrated that axons in α2-chimerin-knockout mice, a loss-of-function model, and mice with knockout of the upstream adhesion molecule, EphA4, exhibited the wandering phenotype in abducens nerves as also detected in our *DINE* mutant mice. These two similar phenotypes suggest the possibility that DINE might contribute to proper axon guidance of abducens nerves via directly affecting the signaling pathway from EphA4 to α2-chimerin.

A previous clinical study reported a missense mutation c.1819G > A (p.G607S) in the *ECEL1* gene as a causal mutation of congenital contracture syndromes, as their in silico analysis predicted the amino acid change to be damaging for the function of the ECEL1 protein [30]. In order to validate and further explore the functional consequences, we generated a *DINE* knock-in mouse with the c.1819G > A (p.G607S) mutation and unexpectedly found a drastic reduction of DINE expression at the transcriptional level, probably via an abnormal splicing process. Recently, it has been revealed that human exons function as sequences necessary for correct splicing in addition to coding information, and disruption of the splice regulatory information in exons leads to pathogenic outcomes [32, 33]. It is often hard to precisely evaluate how identified disease-causing missense mutations affect the splicing process, mainly due to the functional overlap between protein-coding sequences and the splice regulatory information [28], but the rapid progress in development of in silico tools to identify the disruption of critical information for correct splicing sometimes enables prediction of the possible mechanism. In fact, one commonly used prediction tool [5] identified that the c.1819G > A (p.G607S) mutation inhibits the binding of a splicing enhancing factor on exonic sequences (Additional file 4: Figure S4). These results emphasize the importance of in vivo characterization with relevant animal models, in addition to in silico analyses, to precisely identify the biological basis of pathogenic missense mutations.

Our biochemical and immunohistochemical analyses provided the first evidence that C760R mutant protein accumulates in neuronal cell soma, possibly due to the loss of appropriate protein conformation and subsequent impairment of protein transportation into axons. The newly generated knock-in mouse with an artificial mutation (C760G) in which arginine was replaced with glycine reproduced the result, supporting the idea that altered localization of C760R mutant protein could result from the loss of a disulfide bond, as a previous study showed for the homologous cysteine in a family member protein [19]. In contrast to the present data, previous cell culture experiments could not detect altered localization of the mutant protein and suggested that the pathogenic mutation possibly affected enzymatic activity [8]. This discordance might depend on the different experimental paradigms used in the previous study and in ours: whereas the previous study overexpressed the mutant protein in non-neuronal cells, we explored the targeting of the endogenous mutant protein in an in vivo situation, i.e. in mouse spinal motor neurons. Although our in vivo analyses strongly suggest that the mutation affects the targeting of ECEL1/DINE, because of the lack of an established activity evaluation system we cannot exclude the possibility that the mutations also affected enzymatic activity. To date, no physiological substrates of ECEL1/DINE have been identified, but recent in vivo overexpression experiments with *DINE*-deficient mice have shown that mutation of the predictive protease domain of the DINE protein failed to rescue the motor innervation defects resulting from deficiency of *DINE* [20]. Identification of the physiological substrates of ECEL1/DINE and subsequent development of an in vitro enzymatic assay are needed to further explore the functional consequences of the pathogenic mutations.

## Conclusion

Based on analyses of clinically relevant mouse models, we suggest that impaired axonal arborization of spinal motor nerves, as well as axon guidance defects in abducens nerves, are common primary causes in two distinct *ECEL1*-mutated congenital contracture disorders. Another important finding was that the functional consequences of the two pathogenic mutations differed: G607S and C760R led to a drastic reduction of DINE expression and altered localization of DINE protein, respectively. Further in vivo experimental studies with the relevant animal models, working in tandem with clinical genetic studies, would contribute to elucidating the pathogenic mechanisms of *ECEL1*-mutated congenital contracture disorders, including DA and CCDD.

## Additional file

Additional file 1: Figure S1. Impaired axonal arborization in homozygous G607S mutant diaphragm. Whole-mount immunostaining of E17.5 diaphragm muscles with anti-GFP antibody. Axonal arborization defects are detected in the homozygous G607S mutant muscles (b, c) but not in the wild-type muscle (a). (DOCX 401 kb)
Additional file 2: Figure S2. Partial splicing defect in heterozygous G607S mutant hypothalamus. (a) Wild-type (*n* = 3) and heterozygous mutant (*n* = 5) DINE transcripts from adult hypothalamus were evaluated by RT-PCR. The arrow and arrowhead indicate the size of the pre-mRNA and mRNA products, respectively. (b) The ratio of band intensity of mRNA and pre-mRNA was significantly decreased in heterozygous G607S mutant mice. Two-tailed Student’s t test, ***p* < 0.01. (DOCX 101 kb)
Additional file 3: Figure S3. Loss of posttranslational modification in C760R mutant protein. Western blotting analysis with glycosidase-digested protein samples from wild-type and homozygous C760R mutant embryos. In contrast to wild-type samples, only a single band could be detected in Endo H-digested mutant samples. (DOCX 74.5 kb)
Additional file 4: Figure S4. In silico prediction for binding sites of splicing enhancing factors. Binding sites of splicing enhancing factors were analyzed using ESEfinder program (release 3.0). One SRSF1 binding site is specifically disrupted in c.1819G > A (p.G607S) mutation in both mouse and human. (DOCX 34.9 kb)
