# Supplementary material for: Distinct functional consequences of ECEL1/DINE missense mutations in the pathogenesis of congenital contracture disorders
Source: Acta Neuropathol Commun. 2017 Nov 13;5:83. doi: 10.1186/s40478-017-0486-9 (PMC5683451; doi:10.1186/s40478-017-0486-9)
Supplement: Additional file 1: Figure S1. — Impaired axonal arborization in homozygous G607S mutant diaphragm. Whole-mount immunostaining of E17.5 diaphragm muscles with anti-GFP antibody. Axonal arborization defects are detected in the homozygous G607S mutant muscles (b, c) but not in the wild-type muscle (a). (DOCX 401 kb) [file 40478_2017_486_MOESM1_ESM.docx]

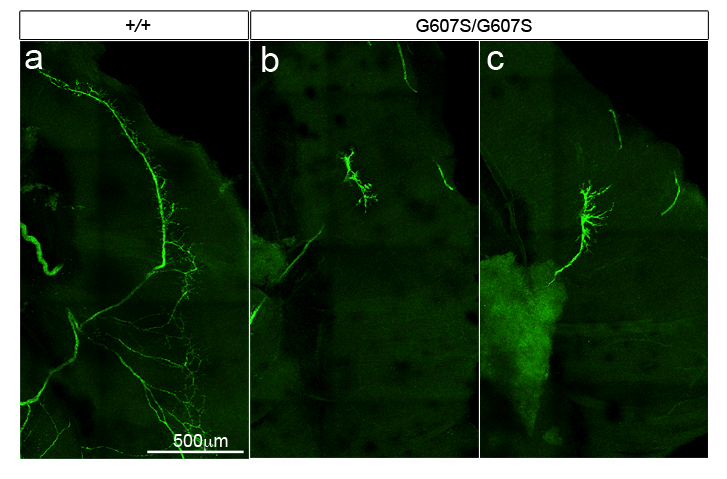
**Figure S1** Impaired axonal arborization in homozygous G607S mutant diaphragm.

Whole-mount immunostaining of E17.5 diaphragm muscles with anti-GFP antibody. Axonal arborization defects are detected in the homozygous G607S mutant muscles (b, c) but not in the wild-type muscle (a).
