# Supplementary material for: Distinct functional consequences of ECEL1/DINE missense mutations in the pathogenesis of congenital contracture disorders
Source: Acta Neuropathol Commun. 2017 Nov 13;5:83. doi: 10.1186/s40478-017-0486-9 (PMC5683451; doi:10.1186/s40478-017-0486-9)
Supplement: Additional file 2: Figure S2. — Partial splicing defect in heterozygous G607S mutant hypothalamus. (a) Wild-type (n = 3) and heterozygous mutant (n = 5) DINE transcripts from adult hypothalamus were evaluated by RT-PCR. The arrow and arrowhead indicate the size of the pre-mRNA and mRNA products, respectively. (b) The ratio of band intensity of mRNA and pre-mRNA was significantly decreased in heterozygous G607S mutant mice. Two-tailed Student’s t test, **p < 0.01. (DOCX 101 kb) [file 40478_2017_486_MOESM2_ESM.docx]

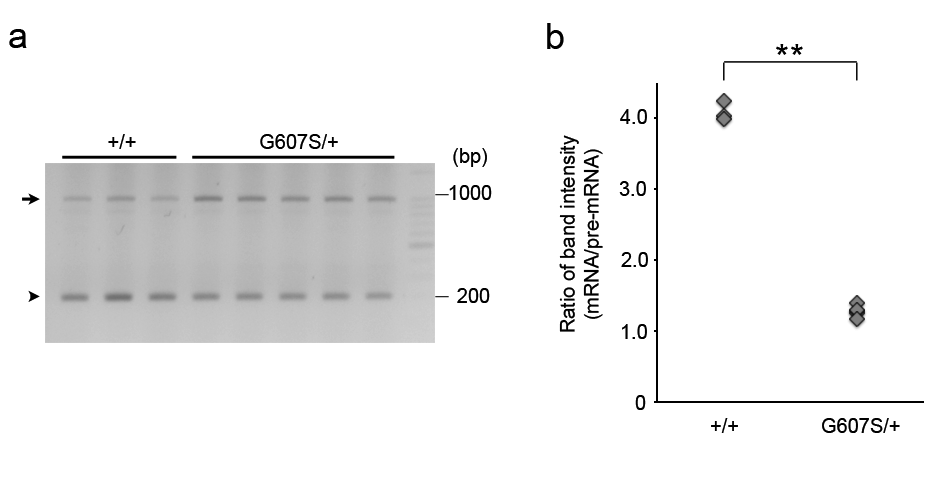
**Figure S2** Partial splicing defect in heterozygous G607S mutant hypothalamus.

(a) Wild-type (*n* = 3) and heterozygous mutant (*n* = 5) DINE transcripts from adult hypothalamus were evaluated by RT-PCR. The arrow and arrowhead indicate the size of the pre-mRNA and mRNA products, respectively. (b) The ratio of band intensity of mRNA and pre-mRNA was significantly decreased in heterozygous G607S mutant mice. Two-tailed Student's t test, ***p* < 0.01.
