# Supplementary material for: Distinct functional consequences of ECEL1/DINE missense mutations in the pathogenesis of congenital contracture disorders
Source: Acta Neuropathol Commun. 2017 Nov 13;5:83. doi: 10.1186/s40478-017-0486-9 (PMC5683451; doi:10.1186/s40478-017-0486-9)
Supplement: Additional file 3: Figure S3. — Loss of posttranslational modification in C760R mutant protein. Western blotting analysis with glycosidase-digested protein samples from wild-type and homozygous C760R mutant embryos. In contrast to wild-type samples, only a single band could be detected in Endo H-digested mutant samples. (DOCX 74.5 kb) [file 40478_2017_486_MOESM3_ESM.docx]

**
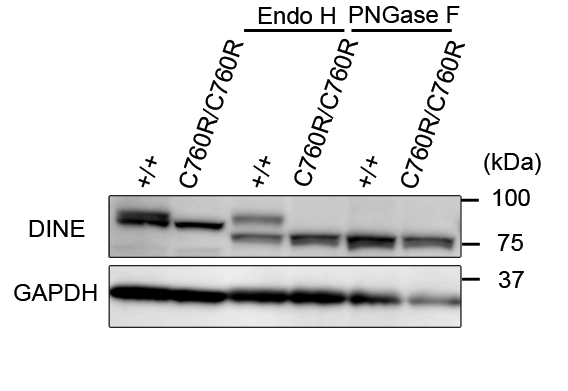
**

**Figure S3** Loss of posttranslational modification in C760R mutant protein.

Western blotting analysis with glycosidase-digested protein samples from wild-type and homozygous C760R mutant embryos. In contrast to wild-type samples, only a single band could be detected in Endo H-digested mutant samples.
