# Supplementary material for: Distinct functional consequences of ECEL1/DINE missense mutations in the pathogenesis of congenital contracture disorders
Source: Acta Neuropathol Commun. 2017 Nov 13;5:83. doi: 10.1186/s40478-017-0486-9 (PMC5683451; doi:10.1186/s40478-017-0486-9)
Supplement: Additional file 4: Figure S4. — In silico prediction for binding sites of splicing enhancing factors. Binding sites of splicing enhancing factors were analyzed using ESEfinder program (release 3.0). One SRSF1 binding site is specifically disrupted in c.1819G > A (p.G607S) mutation in both mouse and human. (DOCX 34.9 kb) [file 40478_2017_486_MOESM4_ESM.docx]

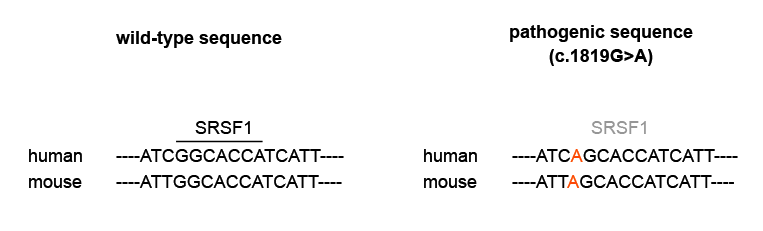


**Figure S4** *In silico* prediction for binding sites of splicing enhancing factors.

Binding sites of splicing enhancing factors were analyzed using ESEfinder program (release 3.0). One SRSF1 binding site is specifically disrupted in c.1819G>A (p.G607S) mutation in both mouse and human.
